# The role of teacher feedback on students’ motivation: a situated expectancy-value perspective

**DOI:** 10.3389/fpsyg.2026.1835077

**Published:** 2026-06-15

**Authors:** Jiaying Chen, Joseph Haw, Zi Yan, Ronnel B. King

**Affiliations:** 1 Department of Curriculum and Instruction, The Education University of Hong Kong, Tai Po, Hong Kong SAR, China; 2 Xavier School, San Juan, Metro Manila, Philippines; 3 Department of Curriculum and Instruction, The Chinese University of Hong Kong, Hong Kong, Hong Kong SAR, China

**Keywords:** feedback, reading performance, situated expectancy-value theory, academic self-concept, learning enjoyment

## Abstract

Teacher feedback has been argued to enhance students’ learning. Recent studies, however, have shown mixed findings. Some studies reported positive, others negligible, and still others a negative impact of teachers’ feedback on students’ learning achievement. To clarify these ambiguities, research is needed to understand the motivational mechanisms through which teacher feedback shapes students’ learning. Through the lens of the Situated Expectancy-Value Theory (SEVT), this study examined whether and how expectancy and value beliefs mediated the relationship between teacher feedback and learning performance. We conducted a structural equation modelling analysis, linking teacher feedback, expectancy, value, and students’ performance, using data from 569,311 students from 80 countries. An inconsistency was identified between the direct and indirect effects of teacher feedback on reading performance. Although the direct relationship between teacher feedback and reading performance was negative, the indirect effect demonstrated that teacher feedback might partially enhance performance by boosting students’ expectancy and value beliefs. Our findings shed light on the motivational mechanisms by which teacher feedback shapes academic performance. Feedback is adaptive insofar as it can be leveraged to enhance students’ expectancy and value beliefs, thereby suggesting a potential pathway for the positive indirect effect of feedback on achievement. For teacher feedback to be truly effective, it needs to target the enhancement of students’ motivation, particularly helping students believe that they can succeed, to recognise the importance of what they are learning, and to derive joy from the learning process.

## Introduction

Teacher feedback has been widely touted as a powerful instructional tool to promote students’ learning (e.g., Hattie and Timperley, 2007; Leenknecht et al., 2021; Wiliam and Thompson, 2008). However, some empirical studies have reported counterintuitive findings, showing that teacher feedback does not necessarily guarantee enhancement of learning. For example, Wisniewski et al. (2020) conducted a meta-analysis to examine the effects of feedback on students’ learning-related outcomes. Surprisingly, 17% of the included studies reported a negative impact of feedback on students’ academic-related outcomes. Recent empirical research also demonstrates some ambiguity. Although several studies have reported a positive relationship between teacher feedback and learning performance (e.g., Ahmed et al., 2021; Wang and Zhang, 2020), other studies have shown that feedback had either a non-significant effect or sometimes even a negative effect on students’ learning performance (Burns et al., 2021; Hu and Wang, 2022; Lipnevich et al., 2021; Yan and Chiu, 2023; Yan et al., 2021).

These inconsistent findings point to the need to explore the theoretical mechanisms by which teacher feedback shapes students’ learning performance. By understanding the specific motivational mechanisms through which teacher feedback facilitates learning, researchers can perhaps disentangle the ambiguities in the existing literature.

We draw on the Situated Expectancy-Value Theory (SEVT) to understand the motivational mechanisms by which feedback shapes learning performance (Eccles and Wigfield, 2020). SEVT foregrounds the importance of contextual factors in shaping students’ learning performance via motivational factors. According to SEVT, teacher feedback could be understood as part of the students’ learning context. To the extent that teacher feedback fosters students’ expectancy and value beliefs, learning would be enhanced. In other words, the effectiveness of teacher feedback could hinge on how students gain better confidence in their skills (i.e., expectancy) and how they can see schoolwork as more enjoyable, relevant, and useful (i.e., value). Elucidating such mechanism demands a comprehensive analysis of the interplay between feedback, motivation, and learning outcomes. Exploring the mediating role of students’ motivation between teacher feedback and their learning outcomes also aligns with a recent trend regarding the agentic role of students in the feedback process (e.g., Carless and Boud, 2018; Winstone et al., 2017).

Previous studies, however, have mostly examined these variables in isolation. Some studies have focused on the association between teacher feedback and students’ motivation (e.g., Ahea et al., 2016; Ani, 2019; Pat-El et al., 2012; Turda et al., 2021). Other studies have focused on teacher feedback and students learning outcomes (e.g., Alharbi, 2022; Burns et al., 2021; Wang and Zhang, 2020) or students’ motivation and students’ learning outcomes (e.g., Li J. et al., 2023; Li Q. et al., 2023; Liu et al., 2012; Lo et al., 2022; Nur’aini et al., 2020). There is a dearth of studies that aim to integrate these distinct theoretical strands to form a more comprehensive understanding of how teacher feedback influences students’ performance through indirect psychological mechanisms.

Hence, the aim of this study is to elucidate the motivational mechanisms through which teacher feedback affect students’ learning. This could answer one of the conundrums in the literature about the reported negligible or negative impact of teacher feedback on students’ learning performance. Adopting SEVT as the theoretical framework to bridge the strands of studies about feedback and motivation together is advantageous and could contribute to two underexplored areas. Firstly, it allows us to delve into the motivational mechanisms through which teacher feedback affect learning. Secondly, it enriches SEVT research by highlighting the importance of context, particularly in the form of teacher feedback. Integrating these two strands of research could also enable teachers to gain a deeper understanding of their students, thereby supporting their individual learning needs more adaptively with refined instructional practice.

## Literature Review

### Teacher feedback and learning performance

The pivotal role of teacher feedback has been documented across numerous studies (e.g., Butler and Winne, 1995; Kluger and DeNisi, 1996; Nicol and Macfarlane-Dick, 2006; Wiliam and Thompson, 2008; Zeng et al., 2018). Teacher feedback operates through a two-step process: firstly, teachers identify and articulate students’ learning gaps, the differences between students’ current understanding and their intended learning objectives, offering them explicit paths for improvement (Martin, 2013). Secondly, students acknowledge their learning gaps, they might utilise them as references to understand better their current learning progress (Denton, 2014; Harris et al., 2014), self-regulate more (Mežek et al., 2022) and have clearer minds to decide further improving actions. As such, the theoretical foundation of teacher feedback to scaffold students’ learning growth is quite powerful.

Though it could be powerful, teacher feedback does not necessarily guarantee students’ learning improvement in practice if students’ perspectives or beliefs of feedback are ignored (Yang et al., 2021). For example, in the domain of reading, some studies reported that teacher feedback had negligible (Swart et al., 2022; Yan and Chiu, 2023; Golke et al., 2015), or even worse, had a negative impact (Bozkurt, 2022; Hu and Wang, 2022; Kluger and DeNisi, 1996; Yan et al., 2021) on students’ performance.

Researchers have proposed viable explanations for these counterintuitive findings. For example, some scholars claimed these findings to be a manifestation of the low-quality of teacher feedback (Shute, 2008; Wu and Schunn, 2020; Yan and Chiu, 2023). Others have argued that this is a spurious correlation that in reality reflects a tendency for teachers to provide more feedback to comparatively low-performing students (Bozkurt, 2022; Hu and Wang, 2022; Yan and Chiu, 2023). Hence, feedback might seem to be negatively correlated with performance not because it leads to poor performance per se but just because low-performing students receive more remedial feedback from their teachers.

It is also possible that there are potential variables mediating the relationship between teacher feedback and students’ learning performance. For example, Zhu et al. (2024) found that teacher feedback does not possess a direct impact on students’ writing proficiency. Instead, they observed an indirect influence on students’ writing proficiency through the mediation of writing self-regulation strategies.

Drawing from feedback theories, motivation is frequently cited as playing a key mediating role among these potential variables (e.g., Carless and Boud, 2018; Kluger and DeNisi, 1996; Lipnevich et al., 2016; Narciss, 2008; Panadero and Lipnevich, 2022). However, empirical studies which explored the mediating role of motivation between teacher feedback and learning performance are limited (e.g., negative affect triggered by teacher feedback surprisingly assisted students in improving essays’ grades, Lipnevich et al., 2021; students who perceived more teacher feedback tended to enjoy reading tasks more, which in turn led to better reading performance, Ma et al., 2023). Given the relative scarcity of these studies, the linkage between teacher feedback and motivation remains relatively underexplored.

Tricomi and Depasque (2016) once proposed two main functions of teacher feedback, namely informative (which aligns more with the aforementioned learning-gap-communicating function of teacher feedback), and motivational, suggesting that teacher feedback could also serve to motivate students’ learning. The motivational aspect of teacher feedback has been empirically verified by neuroscientific research (Mangels et al., 2006; Ng, 2018; Tricomi and DePasque, 2016). Given the propositions of these feedback theories and the relevant studies as a foundation, when investigating the inconsistent findings regarding the impact of teacher feedback on students’ learning performance, motivation becomes a crucial factor that merits consideration.

### Situated expectancy-value theory

Situated Expectancy-Value Theory (SEVT) (Eccles and Wigfield, 2020) serves as an overarching theoretical framework to clarify the ways in which teacher feedback, via motivation, leads to student learning outcomes. Though SEVT is rooted in the influential Expectancy-Value Theory (EVT) (Eccles et al., 1983; Wigfield and Eccles, 2000), it extends from it by focusing on the impact of contexts on students’ achievement-related choices and outcomes through the two proximal psychological determinants, expectancy and value (Li J. et al., 2023; Li Q. et al., 2023; Sun et al., 2023). Contexts, according to Eccles and Wigfield (2020), are constituted of different factors, including cultural (e.g., cultural milieu), historical (e.g., previous experience) and social (e.g., socialisers’ behaviours) factors. Essentially, students are more likely to achieve better learning outcomes when they are situated in contexts where cultural, historical, or social factors positively influence their motivation.

In school, teachers are students’ key socialisers. Some studies have examined how teachers’ hehaviour such as instructional styles (Schweder and Raufelder, 2022) and different support (Rubach et al., 2023) influenced students’ expectancy and value. Similar to other teacher behaviours, it is presumable that when teacher feedback enhances students’ expectancy and value, it could lead to better student learning performance. However, teacher feedback, despite being a critical instructional behaviour from a key socialiser, is surprisingly underexplored in the SEVT framework.

Expectancy and value are two complex and domain-specific psychological determiners (Eccles and Wigfield, 2020). Expectancy pertains to belief that one can do it and value pertains to the belief that what one is doing is enjoyable and important. Existing studies usually concretised them into more measurable constructs, such as domain-specific self-concept for expectancy (e.g., Lauermann et al., 2017; Locher et al., 2021) and domain-specific enjoyment for value (e.g., Geng et al., 2022; Li J. et al., 2023; Li Q. et al., 2023).

Research has established associations between teacher feedback and these domain-specific constructs of expectancy and value. Specifically, teacher feedback plays a role in enhancing students’ expectancy. For example, students who perceived positive feedback about their abilities showed an increase in their self-concept in reading (Burnett, 2003). Positive academic feedback was also linked to a higher level of academic self-concept (Chen et al., 2011). More recently, a correlation between adolescents’ feedback seeking and their positive self-concept of ability was identified (McPartlan et al., 2021). In the domain of reading, exploring data from PISA 2018, Ma et al. (2022) reported a positive correlation between students’ perception of teacher feedback and their reading self-concept across the selected collectivistic and individualistic countries.

Moreover, teacher feedback also plays a role in promoting not only the levels but also the scope of students’ subjective value towards learning. For instance, a positive relationship between middle-school students’ perception of positive general feedback and their enjoyment was reported in the physical classroom (Koka and Hein, 2003). Corrective feedback was found to expand the scope of students learning enjoyment in the language learning classroom (Bayat et al., 2020). Ma et al. (2023) further extended their findings by analysing the large-scale data from PISA 2018. Among all 75 countries/economies, a student-level positive association between teacher feedback and students’ reading enjoyment was identified in 73 of them.

### Motivation and learning performance

Motivation could be broadly defined as a psychological state that energises, directs, and sustains behaviour (Eccles and Wigfield, 2020; Ryan and Deci, 2000). SEVT is one of those theories about motivation that suggests internal pathways from context to behaviour through expectancy and value. Studies have also substantiated the role of domain-specific expectancy and value in shaping students’ learning-associated achievements (e.g., Lauermann et al., 2017; Marszalek, 2022; McKellar et al., 2024; Penk and Richter, 2017), including reading performance (e.g., Cantrell et al., 2018; Cartwright et al., 2016; Griffin et al., 2020; Retelsdorf et al., 2011; Yeung et al., 2022). Specifically, expectancy could exert long-term and short-term affirmative influence on learning achievement. For example, both Chiu and Klassen (2009) and Ma et al. (2021) noted positive correlations between reading self-concept and reading performance with cross-sectional data from PISA. Durik et al. (2006), in contrast, highlighted that students’ English self-concept of ability in Grade 4 forecasted their literacy self-concept of ability in Grade 10, which, in turn, facilitated students’ leisure reading time, course choices in high school and their career aspiration in Grade 12. In efforts to decipher the underlying mechanism between reading self-concept and reading performance, Locher et al. (2021) identified intrinsic motivation as a pivotal variable in elucidating this relationship. Furthermore, academic self-concept showed a significant association with assessment activities (e.g., self-assessment) which, in turn, influence learning performance (Yang et al., 2023).

Research also reveals how value act as a catalyst for learning performance. Lee (2014), Tavsancil et al. (2019), and Ma et al. (2023), for example, all identified a positive association between reading enjoyment and reading achievement, with data from PISA 2009 or 2018. Particularly, in Lee (2014), reading enjoyment was reported as the foremost predictor across 13 eastern and western high performing countries. Similarly, Whitten et al. (2019) discovered that high school students who engaged in reading for personal pleasure scored higher in academic subjects. Others, such as Mucherah and Yoder (2008), probed into a more nuanced form of reading enjoyment, reading for aesthetic enjoyment. Students who read for aesthetic enjoyment outperformed their counterparts in a standardised test.

Furthermore, motivation serves as an essential intermediary between environments around students and their learning process. Existing research on SEVT has also endeavoured to understand how students’ motivation influences the mechanism through which teacher behaviours, as contextual factors, affect students’ learning-related outcomes. For example, Rubach et al. (2023) found that teacher support reduced students’ negative affect in mathematics through their perceived teacher support and interest value. Importantly, not all teacher behaviours result in improved student performance. Schweder and Raufelder (2022) discovered that, teacher-centred instruction seemed to be less effective in boosting student grades in their preferred subjects via increasing students’ expectancies and subjective task value, comparing to student-directed learning. These studies evidence that while teacher behaviours could influence learning outcomes through motivation, this influence could be either positive or negative. This principle should also apply to teacher feedback, which is also a form of teacher behaviour. In fact, in their foundational work about EVT, Eccles et al. (1983) used teacher feedback in their work to demonstrate how socialisers’ behaviour could influence students’ learning and, Muenks et al.’s (2018), which summarised key instructional practices related to SEVT to support students, also highlighted the importance of teacher feedback. More recently, Gan et al. (2021) reported on the mediating effect of students’ feedback motivation and feedback behaviour between teacher feedback and grades of an English course in non-key universities in China. Their study, however, was limited in nature as they only included variables of value (i.e., perceived usefulness and interest in feedback), omitting variables of expectancy, the other proximal psychological determiner in EVT. Furthermore, Gan et al.’s (2021) study was conducted among university students. As students’ expectancy and value profiles are changing over time (Gorges et al., 2018; Guo et al., 2015), their result might not be applicable in school education setting. Taken together, these findings suggest the existence of indirect psychological mechanism between teacher feedback and learning outcomes more comprehensively in school education setting.

### The current study

Therefore, the current study aims to investigate the motivational mechanisms through which teacher feedback shapes learning performance using SEVT as the framework. Specifically, we examined whether and how expectancy and value mediated the relationship between teacher feedback and learning performance. SEVT is adopted as the theoretical framework due to its capacity to accommodate teacher feedback (as a contextual factor), students’ motivation and their academic performance. In particular, the current study focused on the following two research questions:

1. To what extent is teacher feedback associated with students’ learning performance?
2. To what extent do expectancy and value mediate the relationship between teacher feedback and students’ learning performance?

## Method

### Data and sample

We utilised data from the 2018 Programme for International Student Assessment (PISA) organised by the Organization for Economic Cooperation and Development (OECD) in this study. PISA 2018 has more than six hundred thousand nationally representative 15-year-old student participants across 79 countries and regions (OECD, 2021). We excluded countries that had no available data and students with 75% missing data on the substantive variables, limiting our viable sample to 569,311 students (50% Females, 50% Males) nested in 20,597 schools.

### Measures

PISA is a triennial assessment program that measures students’ achievement scores in reading, mathematics, and science, but focuses on one domain in every cycle. The 2018 PISA focused on reading literacy as its main domain of assessment. Aside from its literacy assessment, it also surveyed the participant’s demographic information and other non-cognitive factors associated with reading literacy. In this study, we used the available questionnaire items related to the participant’s perceived teacher feedback, reading enjoyment, and reading self-concept to measure our substantive variables. The full questionnaire items were presented in the Supplementary Files. All items were rated on a 4-point Likert scale (1 – strongly disagree; 4 – strongly agree). The internal reliabilities of all involved scales are found in Table 1.

**Table 1:** Descriptive statistics and bivariate correlations (*n* = 569,311).

Variables	*M*	SD	*α*	Reading performance	Teacher feedback	Reading enjoyment	Reading self-concept	Gender
Reading performance	448.38	107.60						
Teacher feedback	2.42	0.84	0.85	−0.05^***^				
Reading enjoyment	2.66	0.69	0.81	0.19^***^	0.08^***^			
Reading self-concept	2.81	0.63	0.76	0.23^***^	0.16^***^	0.36^***^		
Gender				0.10^***^	−0.06^***^	0.23^***^	0.05^***^	
Socioeconomic status				0.46^***^	−0.02^***^	−0.02^***^	0.14^***^	−0.02^***^

Gender, having a binary value, was recoded to 0 (for males) and 1 (for females). ***p < 0.001.

### Predictor variable

Our study examined the students’ perceived teacher feedback as a predictor. In line with previous PISA studies (e.g., Eryilmaz and Sandoval-Hernandez, 2021; Cai et al., 2024), we operationalised teacher feedback as a single latent variable composed of three items. The question items include “The teacher tells me in which areas I can still improve,” “The teacher gives me feedback on my strengths in this subject,” and “The teacher tells me how I can improve my performance.” All were about improvement-oriented feedback. All these three items refer to information given to students about their performance by teachers, aiming to improve their learning. Students responded to each item on a four-point scale about frequency, from 1 (Never or almost never) to 4 (Every lesson or almost every lesson).

### Mediators

Expectancy (i.e., reading self-concept) and value (i.e., reading enjoyment) were measured as mediator variables. We represented reading self-concept as a single latent variable composed of three items (e.g., “I am a good reader”). We used a five-item questionnaire (e.g., “Reading is one of my favorite hobbies”) to operationalise a single latent variable for reading enjoyment. We reverse-coded the negatively stated items to follow a positive direction.

### Outcome variable

We used the 10 plausible values for reading proficiency in the PISA database to represent reading performance as an outcome variable. These plausible values were not actual scores. Rather, they were imputed, probabilistic estimates randomly drawn from the populations’ ability distribution to indicate the range of possible scores the students could reasonably have achieved in the test based on Item-Response Theory (OECD, 2009). Plausible values are used in large-scale assessments like PISA for group-level inferences, not for individuals. This approach represents measurement uncertainty and provides unbiased estimates of population-level parameters, avoiding the underestimation of variance that would result from using a single estimate per student (Wu, 2005). The scores were scaled to have a mean of 500 and standard deviation of 100 using the Rasch model.

### Covariates

PISA used an index of economic, social, and cultural status (ESCS) as a composite indicator of the individual’s socioeconomic status (OECD, 2019). The index was centred on the OECD mean with a value between −1 and 1. Hence, negative values indicate a composite socioeconomic indicator below the OECD mean, whereas positive values indicate being above the OECD mean. PISA used a binary gender question asking the participants “Are you a male or female?” Several studies have shown that socioeconomic status and gender were highly associated with academic performance (Ghazvini and Khajehpour, 2011; Khesht-Masjedi et al., 2019; Pomerantz et al., 2002). OECD had also observed these associations in PISA 2018 (OECD, 2019). Hence, to isolate the confounding effects of these variables and ensure the robustness of our hypothesised model, we included socioeconomic status and gender as covariates.

### Analytic strategy

#### Preliminary analyses

We employed a multiple imputation method using multivariate imputation via changed equations (MICE) package in R (van Buuren and Groothuis-Oudshorn, 2011). Next, we summarised our data and examined the study variables’ correlation. Following PISA’s analytic method, we used *bifiesurvey package* in R (BIFIE et al., 2022) to include the weights and replicates in computing the statistics. Furthermore, we followed Gignac and Szodorai’s (2016) cut-offs for evaluating individual differences effect size (i.e., 0.10 as small, 0.20 as a typical effect size, and 0.30 as typically large).

#### Primary analyses

We tested our hypothesised model using Anderson and Gerbing’s (1988) two-step approach to structural equation modelling in R *lavaan package* (Rosseel, 2012). The main focus of our investigation was the individual level association of variables. As students were nested within schools, we adjusted the standard errors using the weights and 80 replicates provided by PISA to account for the nested nature of the data (Huang, 2016; Stapleton et al., 2016). To do this, we first refitted the model with PISA weight and weight replicates using R *lavaan.survey package* (Oberski, 2014) before we proceeded with model evaluation.

We first conducted a confirmatory factor analysis (CFA) to examine the model to data goodness of fit. We utilised a robust maximum likelihood estimator as it is designed to handle non-normal data. Expecting that chi-square test will reject our large sample, we relied on other fit indices, as suggested by Hu and Bentler (1995), in evaluating our model: (i.e., CLI/TLI ≥ 0.95 is a good fit; CLI/TLI ≥ 0.90 is acceptable; RMSEA/SRMR ≤ 0.05 is a good fit; RMSEA/SRMR ≤ 0.08 is acceptable). Next, we added the full structural equation model (SEM) and evaluated the fitted model using the same criteria as CFA. We estimated 10 separate models and their respective standard errors for each of the 10 plausible values and aggregated them as a final stage (OECD, 2009).

## Results

### Preliminary analyses

The summary statistics and bivariate correlations are presented in Table 1. Results showed that reading enjoyment and reading self-concept were positively and significantly associated with reading performance with a moderate effect size. Results further suggested that the two mediators are positively correlated with large effect size. However, contrary to what was expected based on prior studies, the results indicated that teacher feedback was negatively associated with reading performance.

Regarding the covariates, both socioeconomic status and gender were significantly associated with reading performance as expected. Specifically, female students and students with higher socioeconomic status performed better in reading than their counterparts. Similarly, the associations between teacher feedback, reading self-concept and reading enjoyment were stronger for female students and students with higher socioeconomic status.

### Primary analyses

CFA results showed a satisfactory model-data fit after allowing two reading self-concept items to covary. As expected, the chi-square statistics rejected the model due to our large sample (Scaled *χ*^2^ = 8936.03, *df* = 40, *p* < 0.001, scaling correction factor = 7.06). However, the other robust-type fit indices indicated that the measurement model fits the data well [CFI = 0.97, TLI = 0.96, RMSEA = 0.05 with 90% Confidence Interval = (0.052, 0.053), and SRMR = 0.04].

Next, we added the structural component. There were 10 models examined as there were 10 plausible values for each variable. All 10 models consistently fit well with the data. Specifically, the fit indices for the model using the first plausible value (PV1READ) indicated an optimal fit [*χ*^2^ = 15252.56, *df* = 66, *p* < 0.001, scaling correction factor = 7.52; CFI = 0.95, TLI = 0.94, RMSEA = 0.05 with 90% Confidence Interval = (0.05, 0.05), and SRMR = 0.04] and the other models followed the same trend. The full results of the individual fit indices are reported in the Supplementary Table S1.

**Figure 1:**
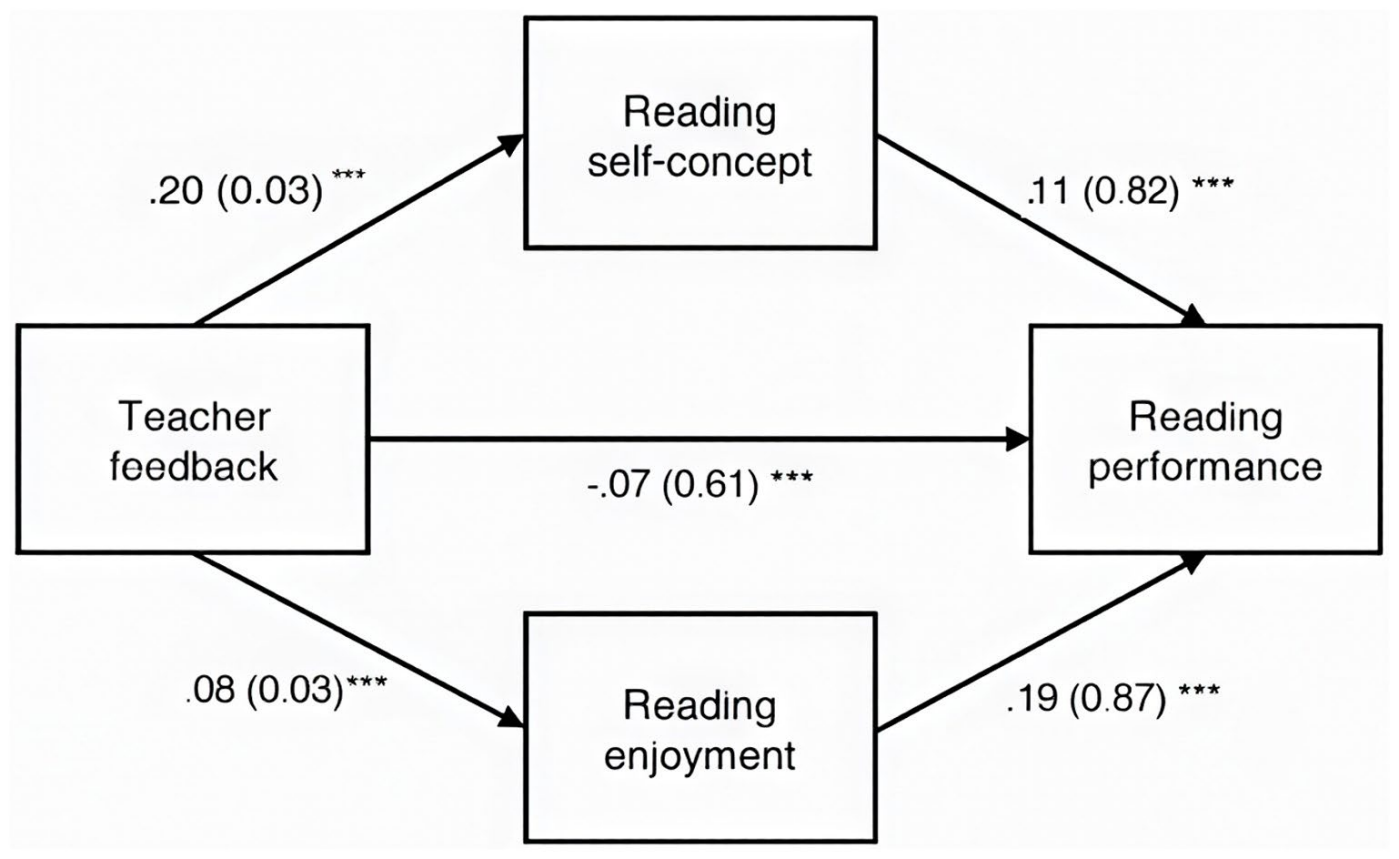
The direct and indirect relationship between teacher feedback and reading performance.

Table 2 shows the final parameter estimates of the associations between teacher feedback, reading enjoyment and reading self-concept, and reading performance after adding the known covariates. Consistent with prior studies, results indicated that teacher feedback is significantly and positively associated both with reading enjoyment and reading self-concept. However, we found that teacher feedback is negatively associated with reading performance. Such association was somewhat counterintuitive but not unexpected as found in earlier PISA research findings (Yan et al., 2021). Nevertheless, results indicated that teacher feedback may have an indirect positive association with reading performance through the mediation of reading enjoyment and reading self-concept, albeit with a small effect size (see Figure 1).

**Table 2:** Structural equation model.

Parameters	Standardised estimates		
	*β*	Gender	ESCS
Teacher feedback→ reading enjoyment	0.08 (0.03)^***^	0.25 (0.03) ^***^	0
Teacher feedback→ reading self-concept	0.20 (0.03) ^***^	0.09 (0.03) ^***^	0.16 (0.02) ^***^
Teacher feedback→ reading achievement	−0.07 (0.61) ^***^	0.05 (0.99) ^***^	0.44 (0.72) ^***^
Reading enjoyment→ reading achievement	0.19 (0.87) ^***^		
Reading self-concept→ reading achievement	0.11 (0.82) ^***^		
Indirect via reading enjoyment	0.02 (0.25) ^***^		
Indirect via reading self-concept	0.02 (0.21) ^***^		
Total effect of teacher feedback and reading enjoyment	−0.06 (0.61) ^***^		
Total effect of teacher feedback and reading self-concept	−0.05 (0.61) ^***^		
*R*^2^ reading enjoyment	0.28		
*R*^2^ reading self-concept	0.07		
*R*^2^ reading achievement	0.07		

^Reading enjoyment and reading self-concept were allowed to be correlated in both models. Estimates in parentheses are standard errors. *** *p* < 0.001.^

## Discussion

Through the lens of SEVT, this study investigated the association between teacher feedback and students’ learning performance (i.e., reading performance), mediated by expectancy (i.e., reading self-concept) and value (i.e., reading enjoyment) using the data of PISA 2018. The result indicated a weak but significantly negative association between teacher feedback and students’ reading performance. While both expectancy and value had a positive impact on students’ reading performance, they also reversed the relationship between teacher feedback and students’ learning performance, somehow transforming it positive (see Figure 1). Given the small beta coefficients, the mediating effect of expectancy and value was positive yet weak. As such, the result of the current study allows us to bridge two strings of studies (i.e., feedback and motivation) together to shed light on how feedback could influence students’ learning through the mediation of motivation.

### Teacher feedback and students’ learning performance

Firstly, a significant and negative association was identified between teacher feedback and students’ reading performance. This finding echoed some past studies (e.g., Bozkurt, 2022; Hu and Wang, 2022; Yan et al., 2021; Wisniewski et al., 2020), again suggesting that teacher feedback does not necessarily guarantee students’ learning performance as theoretically framed. As discussed in the literature review part, current assumptions regarding the negative association are threefold: low-quality teacher feedback, teachers’ inclination to provide more feedback to underperforming students, and the yet unclear process through which students utilise feedback (Yan and Chiu, 2023). Secondly, a small effect size was also observed between teacher feedback and students’ reading performance. This indicated that their relationship might be more indirect, somehow supporting the third assumption.

However, it is also necessary to acknowledge that this negative relationship might be attributable, at least partially, to the measurement properties of the teacher feedback items in PISA 2018. As mentioned previously, the three items operationalised teacher feedback in this study were mainly improvement-oriented. In other words, they captured the type of gap-closing feedback that teachers typically give to students who have not yet met predetermined learning objectives. Consequently, such feedback is disproportionately received by lower-performing students, which might inflate the negative association observed between teacher feedback and reading performance and introduce a degree of ambiguity into the interpretation of the current results. Future studies could benefit from employing more differentiated measures of teacher feedback (e.g., feedback quality, valence, and perceived usefulness) to examine its effects on students’ learning.

### The mediating roles of reading expectancy and reading value in the relationship between teacher feedback and reading performance

As discussed in the literature review part, though motivation theoretically mediates the relationship between teacher feedback and learning performance (e.g., Carless and Boud, 2018; Kluger and DeNisi, 1996; Lipnevich et al., 2016; Panadero and Lipnevich, 2022), scant studies have empirically investigated their validity and how motivation variables influence their indirect relationship. Our investigation takes SEVT as the guiding framework. The results indicate that students’ expectancy and value beliefs mediated the link between teacher feedback and students’ learning performance.

To be more specific, teacher feedback promoted students’ reading performance by enhancing their reading self-concept and reading enjoyment. Not only did our results validate the mediator role of motivational variables in the association between teacher feedback and learning performance, but it also provided some intriguing insights when comparing the inconsistent direct and indirect relationship within this model.

This mediation result demonstrated a consistent pattern with SEVT. According to SEVT (Eccles and Wigfield, 2020), the association between socialisers’ behaviours (e.g., teacher feedback), as a contextual factor, and students’ academic performance is likely to be influenced by students’ expectancy and value beliefs. Drawing upon previous research, this mediation result could be further elucidated. For example, the mediating effect of expectancy might be attributed to variables, such as expectation and self-confidence. Expectations set in teacher feedback led to variations in students’ learning (Eccles et al., 1983). When students perceive higher expectations from their teachers, they tend to exhibit greater confidence in their abilities (Szumski and Karwowski, 2019). This confidence could, consequently, increase students’ active participation in the learning process. Similarly, the mediation effect of enjoyment might be explained by student engagement (Khine et al., 2023; Ma et al., 2023). When teacher feedback contributes to fostering students’ enjoyment related to learning, this positive emotional experience might enhance student engagement (Whitten et al., 2019).

Noteworthily, the beta coefficients for the indirect effects were relatively small. Perhaps, this also suggested the existence of other variables underneath the relationship between teacher feedback and learning performance, which could evidence one of the assumptions previously mentioned in the literature review, attributing the negative association between teacher feedback and learning performance to the unclear process of how students utilise feedback (Yan and Chiu, 2023). In line with this, Panadero and Lipnevich’s (2022) review of feedback models emphasised the critical role of feedback processing, encompassing cognitive, motivational, and metacognitive effects, in determining the outcomes of feedback. Note that in this study, we only focused on the motivational mechanisms. Additionally, students’ willingness and ability to respond to teacher feedback (Tay and Lam, 2022) or student feedback literacy influence the impact of teacher feedback on student learning (Yan and Chiu, 2023).

The small indirect effects might also point to the critical role of student agency in the feedback process. Scholars in this field have increasingly advocated to posit students as proactive participants in their own learning (Carless and Boud, 2018; Winstone et al., 2017), and the present findings are consistent with this perspective. From this point, the effectiveness of teacher feedback is not only determined by its focus, characteristics or frequency; it is also about whether and to which degree students possess feedback literacy to interpret, evaluate, and act upon the information they receive. Students with higher feedback literacy are better equipped to process the improvement-oriented feedback, or any type of feedback, and to translate it into enhanced motivation (e.g., expectancy and value), and eventually into improved learning performance. The findings of this study therefore suggest that interventions aimed at fostering student agency and feedback literacy might be a critical component for effective teacher feedback practices. Given our findings and the propositions put forth by previous research, there remains ample scope for further exploration of the indirect influence of teacher feedback on learning outcomes, especially through cognitive, motivational, and metacognitive factors or mechanisms that related to students themselves.

Although expectancy and value played an approximately equal role in enhancing the relationship between teacher feedback and learning performance, their respective relationships with teacher feedback and learning performance varied. The stronger association between teacher feedback and expectancy implies that it is a more sensitive factor in explaining the variation of feedback. Interestingly, reading value had a more significant impact on learning performance than expectancy. This result is different from previous studies where expectancy was more related to learning outcomes (e.g., Geng et al., 2022; Guo et al., 2015; Eccles and Wigfield, 2002). Our study therefore highlights the need to put more focus on the value dimension of SEVT. This might be especially true for the reading domain, where students have more freedom and autonomy to choose the reading materials they prefer, and the text they will read for personal enjoyment.

Furthermore, it is rather intriguing to observe an inconsistency between the direct effect and indirect effects. With reading enjoyment and reading self-concept as mediators, the direct and negative association between teacher feedback and reading performance could be rendered positive, proposing that teacher feedback might play a double-edged role in students’ learning contexts. From the perspective of SEVT, this could mean that the proximal and distal mechanisms that connect a situational factor and students’ learning could be very complex and distinct, just like what Shin et al.’s (2022) reported in their SEVT related study. Similarly, we also suspect that feedback which solely focuses on its informational function (Tricomi and DePasque, 2016) might be too utilitarian to prompt significant changes in learning, whereas feedback that satisfies both informational and motivational needs (e.g., addressing students’ expectancy and value beliefs) is more likely to yield desirable results.

In Hattie and Timperley’s (2007) review on feedback, they concluded that feedback is about the information that can be used to scaffold students to address learning discrepancy between what they know and what they want to know. However, teacher feedback does not only serve a cognitive function. It can also have a motivational function through stimulating students’ effort, engagement and, motivation to reduce such learning discrepancy.

Applying SEVT as the theoretical framework allows this study to validate whether teacher feedback could fuel students’ motivation and consequently improve learning performance by stringing feedback, motivation and learning performance. This approach allows for a deeper understanding of the operational mechanisms of teacher feedback and provides valuable insights for practical reflection.

In SEVT research, gender and socioeconomic status are also important variables that interplay with socialisers’ behaviour to impose impact on learning (e.g., Lee et al., 2021; Li J. et al., 2023; Li Q. et al., 2023; Schweder and Raufelder, 2022; Wang et al., 2023; Yeung et al., 2022). This study controlled gender and socioeconomic status as covariates in the mediation model. Specifically, the associations between teacher feedback and other variables for female students, including reading expectancy and reading value, and reading achievement, were stronger than those for male students. Students with higher level socioeconomic status perceived a stronger association between teacher feedback and reading self-concept/reading achievement than students with lower ESCS. Such findings were in line with SEVT and echoed previous studies (e.g., Berlin and Dargnies, 2016; Held, 2023; Yeung et al., 2022; Wolters et al., 2014), supporting gender and socioeconomic status as unignorable variables when unpacking the association between teacher feedback and learning performance.

There are viable reasons to explain the roles of gender and socioeconomic status in this association. For example, teacher feedback towards female and male students could potentially display different features (e.g., different levels of expectation, Eccles et al., 1983), given the distinct attributes associated with genders. Also, female students, who are comparatively more sensitive to external information (Chen et al., 2018), possess higher likelihood to value teacher feedback, internalise it and engage in corresponding actions. Meanwhile, research regarding socioeconomic status and education proposes that more advantaged parents might have higher expectation towards their children, involve more in their children’s education and afford more learning resources for them (Yeung et al., 2022), consequently motivating their children to delve further into the learning process and approach teacher feedback with higher seriousness. Different parental involvement patterns relevant to cultural backgrounds (e.g., European American parents prioritising school volunteering while Chinese American parents emphasising home teaching, Huntsinger and Jose, 2009) might influence how students perceive teacher feedback and consequently, their academic performance. Given the multiple effect of gender and socioeconomic status between the association of teacher feedback and learning performance, further investigation to dive deep into the underlying mechanism is needed.

Generally, students’ performance is enhanced when teacher feedback promotes students’ expectancy and value for learning. Moreover, it also sheds light on the operational process through which teacher feedback contributes to learning, providing opportunities for intensive and critical reflection on the practice of teacher feedback.

### Implications

This study has implications for both teacher feedback research and SEVT work. Firstly, current research on teacher feedback’s working mechanisms usually adopts a pedagogical perspective and its underlying motivational mechanisms remain underexplored. The confirmation of the mediating effect of expectancy and value beliefs enables us to develop a more nuanced understanding of the motivational processes that help translate teacher feedback to better learning performance. This finding bridges the gap between research about feedback and motivation, opening new avenues for future research to investigate their indirect psychological process. Further investigation in this area would help better understand the psychological processes associated with feedback and increase the effectiveness of feedback in assisting students’ learning. Secondly, the confirmation of students’ motivation (i.e., expectancy and value in this study) as one mechanism through which teacher feedback influences learning performance also informs instructional practices. Using SEVT as the theoretical lens, it becomes evident that teachers play a pivotal role as important socialisers of students (arguably the most important socialiser in the school setting for the time and connection with students). As such, they bear great responsibility to actively foster positive expectancy and value beliefs among their students through different pedagogical practices, to eventually construct a learning-supportive environment. In other words, to optimise the effect of teacher feedback, the feedback should cater to students’ motivation to learn. To promote students’ expectancy beliefs means to improve their general self-perceived ability to do certain tasks. This is not an easy task for teachers, as students’ expectancy belief does not develop in merely one task. And sometimes, a vicious cycle might emerge unexpectedly, where students with low expectancy belief avoid engaging in a task, leading to their lower performance. Teachers may follow principles suggested by Kim and Bong (2023), namely “guiding students to appraise their confidence toward specific tasks and goals, exposing students to competent models, providing students with credible social persuasion, and cultural influences on the formation of self-efficacy” (p. 219) to cultivate students’ expectancy belief. Teachers can also help students compare their current levels of performance with their prior performance. This is healthier than students comparing themselves with their peers. When students see their learning progress, especially in terms of how much they have improved over time, they are more likely to develop better expectancy beliefs (Capa-Aydin et al., 2018; Hatlevik et al., 2018; Johnston et al., 2021).

Students’ value belief is contingent upon their subjective perception of enjoyment associated with the activities or tasks they undertake. Enjoyment, as a state of positive emotions, has been empirically found to be transferrable from teachers to students during pedagogical practice (Frenzel et al., 2018). In this case, in endeavours to foster students’ value belief, it is crucial for teachers to not only deliver feedback in positive valence. To optimise the transmission of value, teachers could also pay attention to the types of activity (e.g., choosing those they themselves find interesting) and the material (e.g., choosing materials that are close to students’ lives, in which they could find easily find relatedness and interest). Teachers could also help students recognise the relevance of what they are learning to their daily lives or to how what they learn could help them achieve valued future goals. Doing so could enhance students’ value beliefs (Harackiewicz et al., 2016; Priniski et al., 2019; Rosenzweig et al., 2022). Yet, as discussed earlier, teacher-directed efforts alone may not be sufficient; student agency, e.g., feedback literacy, is equally crucial in the feedback process. Accordingly, teachers are encouraged to provide opportunities for students to self-reflect on feedback so as to actively cultivate their agentic dispositions, which are necessary for feedback to enhance their motivation meaningfully.

Teachers should also modify their feedback strategies according to the gender and socioeconomic status of their students. Specifically, they should be more cautious about the valence of feedback when providing feedback to female students, given that they tend to be more sensitive to information other than (e.g., cognitive and affective components) in feedback. Meanwhile, teachers could devote more feedback to disadvantaged students. Their feedback might also need to be more supportive in nature as disadvantaged students are less likely to receive less support from their families. Therefore, additional support from teachers through feedback could be beneficial.

Thirdly, the current study also acknowledges the significance of teacher feedback as a socialiers’ behaviour in school education setting within the framework of SEVT, in a way that complements the findings in Gan et al. (2021). This further highlights an area in SEVT research that has been largely overlooked, where teacher feedback is seldom considered as a contextual factor despite being used as an example to in Eccles et al.’s (1983) original work about EVT to illustrate how teacher expectations contained in teacher feedback influence female and male students differently. Noteworthily, this should be a very first step for feedback study guided by SEVT framework, as how a socialisers’ behaviour contribute to learning is a complex process where other psychological processes such as students’ perception and interpretation pave this road as well (Eccles and Wigfield, 2020). Also, as the range of socialisers’ behaviour is quite broad, the current study merely acknowledges teacher feedback, as one of the most instrumental behaviour of students’ socialisers. To enhance support for students learning, other important contextual factors could be explored to delineate a more comprehensive picture of students’ learning environment.

### Limitations and future directions

Despite the accessibility offered by PISA data for conducting extensive cross-sectional studies in the field of education, it should be noted that this data was collected at a specific point of time. Considering the data’s cross-sectional nature, it could be a bit hasty to conclude causal relationships. To truly see whether the predictive relationships exist, longitudinal or experimental studies should be conducted.

Furthermore, this study adopted predetermined items from PISA. While this study did successfully elucidate the process of how teacher feedback contributes to students’ learning performance through psychological determiners, important components of feedback, such as affective components, were not addressed in the included items and thus, might lead to results that were discrepant with previous studies.

Last, all the data used in this study were based on the quantitative paradigm. It might be useful to triangulate this with qualitative evidence. Specifically, students could be interviewed about their perceptions of teacher feedback and how such feedback shapes their motivation, specifically their expectancy and value beliefs. Doing so could yield a more nuanced picture of how teacher feedback shapes learning outcomes. It might also be able to generate to new discoveries as teacher feedback could shape other types of motivational processes aside from expectancy and value, which we used a-priori in this study.

## Conclusion

The current study revealed that teacher feedback’s influence on students is mostly indirect, and motivation plays an important role in mediating the relationship between feedback and performance. More specifically, teacher feedback seemed to enhance students’ reading performance via promoting their expectancy and value beliefs. Thus, for teacher feedback to be effective and efficacious, teachers should pay attention to how their feedback could lead to more positive expectancy and value beliefs. Our study contributes to the teacher feedback literature by showing that aside from its pedagogical effects, teacher feedback also has important motivational functions.

## Data Availability

The original contributions presented in the study are included in the article, further inquiries can be directed to the corresponding author.
