# Supplementary material for: The role of teacher feedback on students’ motivation: a situated expectancy-value perspective
Source: Front Psychol. 2026 Jun 15;17:1835077. doi: 10.3389/fpsyg.2026.1835077 (PMC13312164; doi:10.3389/fpsyg.2026.1835077)
Supplement: Supplementary file 1 [file Data_Sheet_1.pdf]

Appendix 1: Items from PISA 2018 used in this study

Teacher feedback:

ST104 **How often do these things happen in your <test language lessons>?**

(Please select one response in each row.)

		Never or almost never	*Some lessons*	*Many lessons*	*Every lesson or almost every lesson*
ST104Q02NA	The teacher gives me feedback on my strengths in this subject.	☐01	☐02	☐03	☐04
ST104Q03NA	The teacher tells me in which areas I can still improve.	☐01	☐02	☐03	☐04
ST104Q04NA	The teacher tells me how I can improve my performance.	☐01	☐02	☐03	☐04

Reading self-concept:

ST161 **How much do you agree with the following statements?**

(Please select one response in each row.)

		Strongly disagree	*Disagree*	*Agree*	*Strongly agree*
ST161Q01HA	I am a good reader.	☐01	☐02	☐03	☐04
ST161Q02HA	I am able to understand difficult texts.	☐01	☐02	☐03	☐04
ST161Q03HA	I read fluently.	☐01	☐02	☐03	☐04

Reading enjoyment:

ST160

How much do you agree or disagree with these statements about reading?

(Please take into account diverse kinds of reading material, such as books, magazines, newspapers, websites, blogs, emails…)

(Please select one response in each row.)

		Strongly disagree	*Disagree*	*Agree*	*Strongly Agree*
ST160Q01IA	I read only if I have to.	☐01	☐02	☐03	☐04
ST160Q02IA	Reading is one of my favourite hobbies.	☐01	☐02	☐03	☐04
ST160Q03IA	I like talking about books with other people.	☐01	☐02	☐03	☐04
ST160Q04IA	For me, reading is a waste of time.	☐01	☐02	☐03	☐04
ST160Q05IA	I read only to get information that I need.	☐01	☐02	☐03	☐04
