# Supplementary material for: The role of teacher feedback on students’ motivation: a situated expectancy-value perspective
Source: Front Psychol. 2026 Jun 15;17:1835077. doi: 10.3389/fpsyg.2026.1835077 (PMC13312164; doi:10.3389/fpsyg.2026.1835077)
Supplement: Supplementary file 2 [file Table_1.pdf]

Table S1

Fit Indices of the Structural Model of the 10 Plausible Values for Reading Achievement

Fit Indices	PV1	PV2	PV3	PV4	PV5	PV6	PV7	PV8	PV9	PV10
$\chi^2(df)$	15252.56 (66)	15343.73 (66)	15413.51 (66)	15298.48 (66)	15355.78 (66)	15314.63 (66)	15355.87 (66)	15328.23 (66)	15303.49 (66)	15262.38 (66)
Satorra-Bentler scaling correction factor	7.52	7.49	7.45	7.51	7.50	7.50	7.50	7.50	7.50	7.51
CLI	.95	.95	.95	.95	.95	.95	.95	.95	.95	.95
TLI	.94	.94	.94	.94	.94	.94	.94	.94	.94	.94
RMSEA [90% CI]	.05 [.05, .05]	.05 [.05, .05]	.05 [.05,.05]	.05 [.05,.05]	.05 [.05,.05]	.05 [.05,.05]	.05 [.05,.05]	.05 [.05,.05]	.05 [.05,.05]	.05 [.05,.05]
SRMR	.04	.04	.04	.04	.04	.04	.04	.04	.04	.04
